# EVIDENCE TO SUPPORT A NOVEL CLINICAL CARE PATHWAY FOR GERIATRIC INCONTINENCE SYNDROME IN OLDER WOMEN

**DOI:** 10.1093/geroni/igae098.2050

**Published:** 2024-12-31

**Authors:** Candace Parker-Autry

**Affiliations:** Atrium Health Wake Forest Baptist, Winston-Salem, North Carolina, United States

## Abstract

Geriatric syndromes define complex health conditions that result from the concomitant presence of multiple geriatric impairments in an older adult. Urinary incontinence (UI), an established geriatric syndrome, is unique in that it is present among younger women due to pelvic floor dysfunction, most commonly due to birth trauma or obesity, as well as older women with aging-related changes in skeletal muscle and cognitive health. The latter is a distinct phenotype of UI called female geriatric incontinence syndrome (GIS). Like other geriatric syndromes, GIS is bidirectionally associated with frailty and commonly occurs with concurrent multimorbidity. The combined impact of pelvic floor dysfunction, aging-related impairments in skeletal muscle and/or cognition, and multimorbidity make defining, diagnosing, and treating GIS a complex task. There is a significant body of work describing non-procedure-based treatments for UI with focus on physical therapy/exercise and medications. However, with unhealthy aging skeletal muscles become smaller and weaker, thus pelvic floor muscle function diminishes. This limits the clinical success of physical exercise to treat geriatric UI. Further, multimorbidity and cognitive impairment may limit the efficacy and safety of pharmacologic UI treatments. Based on our teams qualitative studies, women with GIS desire significant improvement in their symptoms; this may be only possible through multi-modal therapy. During this session, we will review the evidence above in more detail and describe the rationale and proposed approach to a multi-modal clinical care pathway, including both conservative and procedure-based treatments, that we are currently implementing for women with GIS at our tertiary care referral center.

